# High-throughput biodiversity analysis: Rapid assessment of species richness and ecological interactions of Chrysomelidae (Coleoptera) in the tropics

**DOI:** 10.3897/zookeys.597.7065

**Published:** 2016-06-09

**Authors:** Jesús Gómez-Zurita, Anabela Cardoso, Indiana Coronado, Gissela De la Cadena, José A. Jurado-Rivera, Jean-Michel Maes, Tinguaro Montelongo, Dinh Thi Nguyen, Anna Papadopoulou

**Affiliations:** 1 Animal Biodiversity and Evolution, Institute of Evolutionary Biology (CSIC-Universitat Pompeu Fabra), 08003 Barcelona, Spain; 2 Herbario y Jardín Botánico Ambiental, Univ. Nacional Autónoma de Nicaragua, León, Nicaragua; 3 Departament de Biologia, Universitat de les Illes Balears, 07122 Palma de Mallorca, Spain; 4 Museo entomológico de León, León, Nicaragua; 5 Institute of Ecology and Biological Resources, Vietnam Academy of Science and Technology (VAST), 18 Hoang Quoc Viet, Ha Noi, Vietnam; 6 Conservation and Evolutionary Genetics, Estación Biológica de Doñana (CSIC), 41092 Sevilla, Spain

**Keywords:** Angiosperms, Biodiversity, Chrysomelidae, insect-plant interaction, molecular ecology, molecular taxonomy, tropics

## Abstract

Biodiversity assessment has been the focus of intense debate and conceptual and methodological advances in recent years. The cultural, academic and aesthetic impulses to recognise and catalogue the diversity in our surroundings, in this case of living objects, is furthermore propelled by the urgency of understanding that we may be responsible for a dramatic reduction of biodiversity, comparable in magnitude to geological mass extinctions. One of the most important advances in this attempt to characterise biodiversity has been incorporating DNA-based characters and molecular taxonomy tools to achieve faster and more efficient species delimitation and identification, even in hyperdiverse tropical biomes. In this assay we advocate for a broad understanding of Biodiversity as the inventory of species in a given environment, but also the diversity of their interactions, with both aspects being attainable using molecular markers and phylogenetic approaches. We exemplify the suitability and utility of this framework for large-scale biodiversity assessment with the results of our ongoing projects trying to characterise the communities of leaf beetles and their host plants in several tropical setups. Moreover, we propose that approaches similar to ours, establishing the inventories of two ecologically inter-related and species-rich groups of organisms, such as insect herbivores and their angiosperm host-plants, can serve as the foundational stone to anchor a comprehensive assessment of diversity, also in tropical environments, by subsequent addition of trophic levels.

## 1. Biodiversity assessment: challenges and approaches

### 1.1. An all-encompassing view on Biodiversity

Few unifying concepts in Biology are so well established and ingrained in scientific and popular thinking as Biodiversity (Wilson 1988). Yet, the actual definition of Biodiversity is as encompassing and universally accepted, as it is elusive or ambiguous. Biodiversity is the diversity of Life, and by diversity of life we can understand every level of organisation, from the structural elements of genes in a particular genome, to the whole biosphere, past and present. The most intuitive idea of biodiversity has its roots in the enlightened and encyclopaedic inventorying efforts that propelled the voyages of discovery in the XVIII^th^ Century to collect and catalogue animals, plants and minerals all over the Globe. This inventorying urge promoted in turn the creation of Museums, Zoos and Botanical Gardens in developed countries, places to keep and share with the public the records of the catalogue (institutions still reputed and alive and experimenting today a renaissance of that cataloguing spirit). Following this tradition, the word biodiversity evokes a display of life forms, or a list or catalogue of species names, ideally ranked following some system. In this context, biodiversity is tightly linked to the practice and development of Taxonomy, after all the science in charge of recognising, describing and naming organisms. Biodiversity inventories thus benefit from every conceptual and methodological advance that has contributed to the maturation of Taxonomy, from the consolidation of evolutionary thinking to the debates on species concepts, from ultrastructural analyses under the microscope to the study of gene differences among individuals or metagenomic analyses of complex environments. However, as we said, the concept of biodiversity is an all-encompassing idea that should reflect any possible way in which life is organised, including supra-organismal assemblages, such as antagonist or mutualistic associations and behaviours, food-webs, communities and biomes, their combination in ecosystems, and so on. This is essentially the diversity of ways in which life forms can interact, an aspect of biodiversity traditionally approached from Ecology, with a boost in recent times thanks to the progress made in the field of community ecology. The diversity of interactions is perhaps a less intuitive idea attached to biodiversity than the composition of a community *per se* (i.e., the idea of the inventory), but both are the complementary angles that shape the all-unifying concept of Biodiversity (Novotny and Miller 2014). Thus, the concept of Biodiversity, certainly the one we will use throughout this assay, merges composition and functioning criteria of diversity in a given environment. These cataloguing and integrative scopes take on their highest relevance when biodiversity assessment is coupled with conservation initiatives, which ideally aim at preserving not only the nominal diversity of life forms but vitally the processes that sustain them too.

### 1.2. The challenge of Biodiversity assessment

When the emphasis of biodiversity assessment focuses on the inventorying angle, this ‘simplified’ view on biodiversity is nonetheless generally restricted by taxonomic expertise, sampling techniques, budgetary limitations, but most of all by the sheer diversity of life forms that even the most simple biomes can harbour. A relatively homogeneous, well-delimited environment, such as a high-mountain lagoon or a monoculture crop, can be home to hundreds or thousands of different species, considering seasonality and transient and resident organisms, particularly when micro-fauna, micro-flora and, needless to say, prokaryotes are taken into account. This situation forces most biodiversity assessment plans to narrow their scope to simplified sampling strategies, e.g. canopy fogging of individual trees or deep-sea or soil probing, and typically to a specific group of organisms or habit, e.g. arthropods, insects, trees, benthic fauna, etc. Inventorying is certainly a challenge, but adding the interactions dimension to biodiversity assessment is nearly utopian. When biodiversity is described considering its functional aspects, it generally requires a much more restrictive assessment, taxonomic and for a particular interaction, e.g. pollinators of a particular plant species, community of animals exploiting a certain tree, or microorganisms with specific bioremediation potential.

These simplified approaches are defensible from an academic point of view, and they are also well adjusted to the serious underfunding for most biodiversity assessment initiatives. However, they are clearly inefficient to tackle the biological, cultural and moral problem dubbed as the Biodiversity Crisis (Western 1992; Singh 2002). Again, the challenge remains the inordinate number of species and the combinatory of their interactions, coupled in great part with the ever-declining expertise in recognising (let alone naming) this diversity. The tip of the biodiversity crisis iceberg are groups of organisms such as angiosperms, birds, amphibians or mammals, amenable to relatively deep biodiversity assessment at least in parts of their ranges, even though the most serious concerns relate to less conspicuous but hyperdiverse groups of organisms, such as the insects (Dunn 2005). Moreover, biodiversity follows gradients, whereby the still highly unexplored tropics show the highest species counts and associations (Pianka 1966; Janzen 1973; Dowle et al. 2013), and for the known fraction of biodiversity, precisely the tropics harbour most of biological diversity perceived under significant threat, the so-called biodiversity hotspots (Myers et al. 2000). For hyperdiverse groups in hyperdiverse regions of the Planet we can generalise that our taxonomic knowledge is basic and our insight into the species ecology is merely anecdotic—although there are, of course, important exceptions (e.g., InBIO Costa Rica; Smithsonian Institution, Barro Colorado, Panama).

The task ahead is titanic. The goal is to unravel the Earth’s biodiversity as fast as possible against the ever-growing extinction rates due to habitat disappearance, fragmentation and alteration, the combined effect of climate change, overexploitation and the impact of biological invasions (Dirzo and Raven 2003; Barnosky et al. 2011). And non-trivially, the challenge is against a worrying cultural trend in this field known as the Taxonomic Impediment, the combined effect of the perception of Taxonomy as an old decaying science and the gradual disappearance of taxonomic expertise (Hebert et al. 2003; Lipscomb et al. 2003; Wheeler et al. 2004). All in all, the task is perhaps unapproachable, a mere intellectual chimera, but the scientific challenge, societal responsibility and achievable benefits are solid reasons to continue investing in biodiversity assessment, improving our assessment potential with training and technical developments.

In recent years, and as a reaction to the biodiversity crisis there has been a proliferation of initiatives aiming at large-scale biodiversity assessment. This is just to say initiatives that aim at a comprehensive (with constraints) characterisation of biodiversity, with a large regional, ecological and/or taxonomic scope. Large-scale biodiversity assessment has been a traditional practice in ecology, particularly in tropical ecology, whereby scientists sample more or less indiscriminately certain environments, providing with thousands of specimens to museums and academic laboratories around the globe. In some cases, specimens are prepared and sorted, becoming amenable for identification and cataloguing when taxonomic expertise is available. However, most typically sorting reaches a relatively high taxonomic rank, too high for meaningful community analyses, and detailed biodiversity assessment stretches indefinitely in time, depending on the interest of experts and accessibility to these collections. Today, large-scale biodiversity assessment, particularly in the context of the race against the doom to extinction of many organisms, is intimately associated to what has been referred to as rapid biodiversity assessment, in other words, quickly collecting information on the species present in a given area (Oliver and Beattie 1993; Basset et al. 1998).

### 1.3. Molecular support to biodiversity assessment

A major boost in rapid and large-scale biodiversity assessment has been possible in the last two decades thanks to the routine implementation of molecular tools as a valuable standard to recognise diversity. The use of DNA for biodiversity assessment has provided with robust solutions for most of the challenges described above. This is a unique character system for all life forms, which is suitable for analysis with standard laboratory methods that require in turn very basic training. Thus, even modest laboratories can engage in the use of this technology for biodiversity assessment without imposing taxonomic restrictions, both in terms of scope and availability of previous knowledge, but also in terms of required taxonomic expertise (Tautz et al. 2003). Also helping the routinely use of these approaches, the cost associated to DNA-based biodiversity assessment keeps dropping as the methods become more efficient and technology less exclusive (Yu et al. 2012). The basic laboratory steps in this procedure fundamentally require the use of DNA isolation techniques, on an individual or environmental basis (e.g., soil sample, residue of filtered sea water, ...), traditionally followed by PCR-based amplification protocols of specific genome regions, *a priori* defined standards for analysis, and finally sequencing of these markers. The process is facilitated because the latter stage can be handed to a profusion of biotechnology companies that offer sequencing services at very competitive prices. Moreover, the innovative boost of sequencing technologies of the past decade, methods collectively known as next-generation sequencing, all free from the limitations of Sanger technology, has facilitated the analysis of environmental samples and little by little displacing the need for an intermediate PCR step in some applications relevant for biodiversity assessment (Timmermans et al. 2010; Zhou et al. 2013; Andújar et al. 2015).

The use of these affordable, classical and revolutionary methodologies can potentially generate uncountable objective data for analysis, huge numbers of nucleotide characters in DNA sequences only limited by the size of the respective genomes involved, whose variability can inform of species diversity in a sample. While data can easily grow to vast amounts, these are nonetheless amenable for study even with modest computational power, given their suitability for large-scale information technology data storage and analyses. Thanks to the incorporation of molecular tools to the toolkit of taxonomists and ecologists, now the challenge and budgetary needs for biodiversity assessment are not anymore on the generation of raw data, but again on the acquisition of samples, on financing fieldwork and expeditions for biological prospection. There is still an important need for specialisation to some extent, in this case to use and develop methods to extract relevant information from collections of DNA sequences for sound biodiversity assessment. Large-scale biodiversity assessment thus rests on a new pillar as important as taxonomy and ecology: bioinformatics. The bioinformatics for biodiversity assessment has experienced an important development, receiving and exploiting the advances of more than half a century of numerical taxonomy and phylogenetics, but also the suitability of DNA sequence data for digital storage and the availability of an ever growing public database for DNA data generated worldwide.

### 1.4. Large-scale DNA-based biodiversity assessment

There are several ways to approach the use of DNA sequences for objective species delimitation and/or identification, but they can be divided fundamentally in two main categories. The first type of approach takes advantage of the easiness for computation of differences among DNA sequences and the assumption of a relatively uniform divergence threshold between intraspecific and interspecific DNA sequence variation. These numerical or phenetic approaches to biodiversity assessment evaluate the match of a sequence of unknown origin against comparable sequence information in a reference database (e.g., via BLAST algorithms; Altschul et al. 1997), or take advantage of more or less sophisticated clustering algorithms to facilitate taxonomic assignment. The most successful initiative following this strategy is the so-called DNA-barcoding (Hebert et al. 2003), which puts the emphasis on species identification. The second type of approaches aims at extracting evolutionary, phylogenetic information from matrices of homologous DNA-sequences to guide species inference (e.g., Wiens and Penkrot 2002). In this case, there are no implicit divergence thresholds, but there is a strong bearing on the concept of monophyly and inference of processes related to the species problem, e.g. gene flow, recombination, incomplete lineage sorting or hybridisation, among others. This field has flourished in the past few years thanks to advances in two areas of research. One is integrative taxonomy (Dayrat 2005; Schlick-Steiner 2010; Andújar et al. 2014), which tries to formalise the procedures to manage multiple sources of data, with a predominant role of molecular data, in defining (and discovering) species. The other encompasses the conceptual and methodological progress on procedures collectively known as species-trees methods, which use coalescence theory to incorporate discordance among multiple gene trees and predict species boundaries (Yang and Ranala 2010; Fujita et al. 2012). In general, phylogenetic methods have found a better use for problems related to species delimitation.

Phenetic approaches are particularly well suited for large-scale biodiversity assessment by virtue of straightforwardness and speed of analysis. However, they have some drawbacks as well. Their hypothetical optimal performance is achieved when there is a complete reference library available for comparisons (Ekrem et al. 2007), and a consistent barcoding gap or species-diagnostic behaviour of the marker of choice (Meyer and Paulay 2005). These criteria may be met for specific groups, but they are not universal. The quality and coverage of reference libraries can improve over time as new data enter the system, but there is a limitation imposed by the Taxonomic Impediment itself in providing solid taxonomies attached to the reference sequences, not to mention the fundamental problem of incompleteness of the inventory of Life. In any case, reliance on a static barcoding gap will always represent a problem, since this is not a universal, intrinsic property of species and DNA data (Meyer and Paulay 2005; Meier et al. 2006). Indeed, some alternatives exist to customise the concept of species thresholds, such as the ABGD method (Puillandre et al. 2012), but there will be always problematic groups for this criterion, e.g. species that hybridise, recent speciation events, convergence and evolutionary stasis or lineage-specific differences in evolutionary rates for the marker of choice. Moreover, taxonomic gaps in the reference library and exceptions to the barcoding gap do not prevent these approaches from producing species inferences even in the absence of true conspecifics in the reference database; these are known as false positives, and constitute one of their most serious limitations (Ross et al. 2008).

In turn, phylogenetic approaches are powerful and can assist both species delimitation and identification when used with a reference. In this case, even if the reference library does not include conspecific data, phylogenetic inference protects against false positives at the expense of taxonomic resolution (Ross et al. 2008; Berger et al. 2011). Phylogenetic theory and practice have pushed dramatic advances in speed of analysis, both with more efficient and faster algorithms and a better use of computing capacities with parallelisation of complex calculations. However, these methodologies tend to be complex analytically, intense computationally and generally benefit from studying multiple markers, therefore are slower, less intuitive and need more training than their phenetic counterpart. Moreover, the performance of phylogenetic inference varies depending on the markers and underlying assumptions, which advises against blind attempts to conduct biodiversity assessment, without a way to evaluate systematically the robustness of the phylogenetic trees.

Clearly, DNA-based biodiversity assessment in the context of large-scale studies, can benefit of tree-based approaches taken from the field of molecular systematics, but it also requires speed of analysis. Specifically related to the problem of species identification, bacterial molecular taxonomy and current efforts to characterise microbiotas in multiple environments (e.g., Human Microbiome Project or TerraGenome) have built upon this tree-based concept for many years now. Thus, in this field, researchers exploit fast maximum likelihood phylogenetic analyses of query prokaryote 16S sequences against curated taxonomic references for this marker, e.g. workbench of Greengenes, SILVA and others (McDonald et al. 2012). Inspired by the philosophy of bacterial taxonomy, we have recently developed an analogous strategy for any kind of organism adding flexibility for the marker of choice by exploiting real-time taxonomically-tagged sequence availability in public nucleotide sequence databases [see section 2.3], the so-called BAGpipe protocol originally applied to angiosperm identification based on *psbA-trnH* data (Papadopoulou et al. 2015).

## 2. The contribution of Chrysomelidae to a diverse world

### 2.1. Leaf beetle communities matter to large-scale biodiversity assessment

The field of conservation biology has relied on bioindicators to monitor the quality of the environment (Noss 1990; Caro and O’Doherty 1999). Rather than attempting massive biodiversity studies on particular environments, perhaps a sound way to enhance biodiversity assessment could find inspiration in the notion of indicators, assessing the biodiversity of certain communities both in terms of taxonomic diversity and their species interactions. The focus would be on a highly diverse group of organisms in a given environment with a range of diverse but representative ecological interactions. Biodiversity assessment on such a group would serve as scaffold to anchor successive complementary studies above and below that particular interaction level, aiming with time at a multitrophic level description of the whole system. In this respect, for terrestrial ecosystems, ubiquitous herbivore insects constitute an excellent focal group to launch large-scale enquiry on the biodiversity and interactions of biomes (Stork and Habel 2014). In our opinion, phytophagous beetles, and leaf beetles in particular, represent a study system with important advantages. Their taxonomic diversity and that of their food-plants can be staggering in any given tropical environment (Erwin 1982; Wagner 1999; Novotny and Miller 2014), in general they portray a tight ecological relationship with plants in all life cycle stages, and have high endemicity rates, both factors generating a perception of strong relationship with the environment. All in all, by focusing on the inventory and interactions of leaf beetles, it is possible to design research simultaneously on two highly diverse components (=indicators) of biota from most tropical ecosystems—insects and plants—, as well as on one of the predominant ecological interactions, herbivory (Price 2002).

Over the past few years (since 2007) we have thus developed on the notion that we can significantly contribute to an enhancement of biodiversity studies by targeting the fast characterisation of complex leaf beetle (or other herbivore insects) communities in the tropics as well as their ecological associations by using a combination of DNA-barcodes, tree-based species delimitation and forensic characterisation of food plants, with a robust and automatable analytical set-up. As a general proposition, we advocate that, when attempting large-scale biodiversity studies, where both delimitation and identification of species represent a challenge, the most efficient approach involves the use of DNA sequence data (only one or few ‘barcodes’) and phylogenetic approaches. Thus, our general workflow for large-scale biodiversity assessment of tropical leaf beetle communities includes four distinctive stages: (1) indiscriminate sampling of chrysomelid beetles in a particular environment or region; (2) non-destructive DNA extractions and specimen preparation for future reference; (3) DNA sequencing of at least one beetle mtDNA marker (typically *cox1*) and at least one putative diet marker (either *trnL* or *psbA-trnH*); and (4) phylogenetic inference for beetle species delimitation and host-plant identification.

### 2.2. Species delimitation and enhanced species discovery

We mentioned above that DNA-enhanced species delimitation has achieved fundamental progress over the past few years in great part thanks to the development of powerful phylogenetic methodologies to deal with gene tree incongruence as well as conceptual advancement on how to integrate taxonomically relevant data. However, these procedures are time and resource consuming, benefiting from the analysis of multiple genes and generally from a good taxonomic knowledge of the group of interest. These tree-based procedures find a good use in systematic research but are impractical for large-scale, rapid biodiversity assessment. Instead, our methods of choice, with a good trade-off between economy and speed of analysis (including data acquisition) and robustness and accuracy of results are the Generalized Mixed Yule-Coalescent model (GMYC; Pons et al. 2006; Fujisawa and Barraclough 2013) and the Bayesian implementation of the Poisson tree processes model for species delimitation (bPTP; Zhang et al. 2013). These are tree-based methods that do not require previous knowledge of species boundaries, making them suitable for the analysis of groups with poor taxonomy, and are specifically designed to work with single locus data (e.g., a DNA-barcode). For example, GMYC tests changes in branching rates at the species boundary on an ultrametric tree based on the optimisation of a likelihood function with predictions for branching patterns both in speciation and population neutral coalescent processes. In practice, the algorithm scans two types of information on gene trees—waiting times between successive branching events and number of lineages within each interval—to optimise a single or multiple thresholds defining species branches on the tree subtending one or more populations evolving under neutral coalescent diversification processes. bPTP in turn relaxes the need for an ultrametric tree and infers species boundaries based on the so-called Poisson tree processes model (Zhang et al. 2013). Focusing on a single standard DNA-barcode lowers the cost and increases the speed and robustness of data acquisition, and both algorithms are fast and accessible thanks to functions of the R package ‘splits’ (SPecies LImits by Threshold Statistics; Ezard et al. 2013) in the case of GMYC, and a fully functional web server (http://species.h-its.org/ptp/) in the case of bPTP, both desirable characteristics for rapid biodiversity assessment.

The suitability of this approach to investigate well-known leaf beetle communities in temperate regions has been shown recently (Baselga et al. 2015). In addition, we are successfully applying it to several projects studying leaf beetle biodiversity at large in different tropical systems for which there is a deficient taxonomic knowledge on the composition of their respective leaf beetle communities. One such study focuses on the diversity of Eumolpinae in New Caledonia, a group that recent taxonomic work has exposed as highly diverse without a precise estimate of the expected total diversity (Gómez-Zurita 2011; Papadopoulou et al. 2013). In other studies we investigate the communities of leaf beetles in dry tropical forests of Nicaragua and Vietnam with a common aim of evaluating biodiversity parameters that can be eventually used for conservation initiatives targeting this highly threatened tropical biome (Janzen 1988; Miles et al. 2006). In these studies, we sampled hundreds of leaf beetle specimens which were individually characterised for one mtDNA standard locus, an 830 bp fragment of the 3’-end of the first subunit of the cytochrome *c* oxidase (*cox1*), and an additional mtDNA locus in the case of New Caledonian Eumolpinae (a 515 bp fragment of the small rRNA subunit, *rrnS*). In every case, the individuals characterised from a genetic viewpoint were preserved and mounted dry, with their genitalia dissected. Vouchering specimens from such large-scale biodiversity studies is essential for fulfilling the inventorying angle of biodiversity assessment, particularly when the lack of readily available taxonomic expertise or the weak taxonomic knowledge of the focal group, hampers the immediate naming of species. The amount of new species for Science in understudied tropical faunas can be high, and subsequent in-depth taxonomic work to name species usually reveals undescribed diversity. As will be seen below, the non-destructive treatment of samples is crucial to allow for species descriptions and instantly provides with standard type material (besides the DNA sequences used to speed up their discovery). Preparation of our processed specimens has yet another short-term practical advantage, which is allowing for a fast complementary assessment of species diversity based on the concept of morphospecies, i.e. groups of individuals that look alike. A comparison between the two pragmatic strategies for rapid species assessment, DNA-based GMYC-groups *versus* morphospecies, can assist in the evaluation of performance of the first, objective method (Papadopoulou et al. 2013), as well as the discovery of new species, while drawing attention to interesting biological characteristics of the system, particularly if sample metadata is taken into account (e.g., geography, biome, host-plant information, etc.).

The systematic implementation of GMYC species delimitation to each of our datasets produced consistently species counts compatible with estimates based on morphospecies assessment (Table 1), and disagreements revealed in general a better performance of the molecular tree-based strategy. Essentially identical results have been shown and the same perception championed by Tänzler et al. (2011) based on their rapid-biodiversity assessment exercise centred on a single hyper-diverse weevil genus in New Guinea, *Trigonopterus*. Additionally, these authors formally explored a very interesting aspect of rapid species assessment that we also experienced from a pragmatic viewpoint, adding to the value of molecular approaches: DNA-based species delimitation outperforms sorting skills by trained, but non-expert parataxonomists. In our experience, there are always a few cases of morphospecies misplacements that benefit from reassessment *a posteriori* using phylogenetic information. These misplacements are not necessarily the result of real identification difficulties, but could be simply owing to visual memory limitations, when dealing with hundreds, perhaps thousands of specimens belonging to dozens or hundreds of species, in the context of massive sampling in tropical settings. Of course, DNA-based approaches have shown their strength in revealing hidden, cryptic diversity, externally invisible to expert eyes, let alone to rapid sorting for accelerated biodiversity inventories (e.g., *Astraptes*, Prado et al. 2011; Staphylinidae, Thormann et al. 2011). However, there is an additional important advantage of using DNA for species delimitation, somehow tackling the opposite scenario offered by cryptic diversity. This is the opportunity to sort accurately all life-stages (e.g., Ahrens et al. 2007), species with colour polymorphism (e.g., Rugman-Jones et al. 2013) or sexually dimorphic species (e.g., Smith and Brown 2008), i.e. situations that are challenging for morphospecies-based assessment of diversity, while they are rather common in insects, in particular in certain groups such as butterflies and many beetles, including the Chrysomelidae. Our research on tropical leaf beetle communities has provided with examples for each of these advantages, matching larvae and adults of the chrysomeline *Plagiodera septemvittata* Stål in Vietnam (Nguyen and Gómez-Zurita, in prep.) or the cassidines *Coptocycla leprosa* (Boheman), *Omocerus caeruleopunctatus* (Boheman) and *Parorectis rugosa* (Boheman) in Nicaragua (Papadopoulou et al. 2015), the very distinctive males and females of several eumolpine species of *Taophila* Heller in New Caledonia (Papadopoulou et al. 2013; Gómez-Zurita and Cardoso 2014), and the highly polymorphic galerucine *Cerotoma atrofasciata* Jacoby in Nicaragua.

**Table 1.** Sampling and sequencing effort, and DNA-based species diversity estimates in three large-scale leaf beetle biodiversity studies in the tropics.

Study	N	Geographic scope	Longest transect	Taxonomic rank	DNA-barcode	GMYC species
New Caledonia	840	Grande Terre	400 km	Eumolpinae	*cox1, rrnS*	107 [94-121]^a^
Nicaragua	1270	Pacific and northern provinces	250 km	Cassidinae, Eumolpinae, Galerucinae,	*cox1*	336 [333-347]
Vietnam	494	Núi Chúa Natl. Pk.	5 km	Chrysomelidae	*cox1*	161 [156-165]^b^

a Averaged data from Papadopoulou et al. (2013).

b Taken and averaged from Nguyen and Gómez-Zurita (in prep.)

Once there is a sound estimate of species numbers resulting from a sampling effort of known intensity, it is possible to investigate how representative the measure of biodiversity is of the total expected diversity. For example, we used a strategy based on rarefaction curves representing accumulation of objectively delimited species across sampling events for New Caledonian Eumolpinae to extrapolate the expected total species richness in the studied environments. From our empirical demonstration of slightly over one hundred species in our ensemble sample, we could analytically propose an expected total number of eumolpine species in New Caledonia between 148 and 210, depending on input data and species richness estimator of choice (Papadopoulou et al. 2013). Preliminary data for three Chrysomelidae subfamilies sampled in Nicaraguan dry forests (Eumolpinae, Cassidinae s.l. and Galerucinae s.l.) or the whole Chrysomelidae community in a National Park in southern Vietnam, both analysed using a similar accumulation-curve approaches as in New Caledonia, reveal that our samples may represent between 53–69% of the total leaf beetle diversity in the studied biomes. Thus, a continued sampling effort should recognise in the order of 500-600 Chrysomelidae species in the abovementioned subfamilies in the dry Pacific side of Nicaragua, or the same number of chrysomelids in a 10 sq. km. forest patch across a slight elevation gradient in southern Vietnam.

The experience gained from this type of studies shows that the main limiting factor for robust diversity assessment is obtaining sampling densities representative of the studied environment always, i.e. fieldwork. Once samples are available, laboratory methods can be optimised in weeks or few months, depending on the number of samples used and smoothness of PCR protocols, and a similar or slightly longer time for standardised analytical procedures.

### 2.3. Forensic methods for the analysis of species interactions

We stressed already that there is one quantitative advantage of molecular characters to aid biodiversity assessment: speeding up the rate of species delimitation and also diagnosis. Additionally, these characters have at the same time the potential to contribute an extremely important qualitative advantage: the possibility to investigate complex systems and processed samples, which is the door to community ecology and the study of food-webs. In 2009, simultaneously with the studies of Valentini et al. (2009) and Soininen et al. (2009) and the earlier approach of Matheson et al. (2008), we pioneered the investigation of animal-plant interactions using DNA (Jurado-Rivera et al. 2009). In our approach, conversely to the mainstream DNA-barcoding stance of these contemporaneous and other subsequent studies, one of our main motivations was to extract taxonomically relevant information from processed food in the face of an incomplete reference database, by exploiting molecular phylogenies as the most rigorous and powerful tool for taxonomic assessment.

In most studies that target trophic associations, DNA extraction is directed to the most obvious sources for food DNA, including gut contents and faeces. In our case, and in great part motivated by the special characteristics of our study organism, the starting material is always the whole leaf beetle specimen, generally small enough to fit the tubes used for the DNA extraction procedure. The main idea is that when we obtain DNA from the whole specimen, we indeed mostly retrieve nucleic acids from the beetle species, useful for its genetic characterisation. However, with host DNA, we obtain simultaneously a significant proportion of DNA from organisms onto and into the beetle, therefore representing the ecological interactions it sustains, including DNA from all of its symbionts, endosymbionts, phoretics, commensals, parasites, hyperparasites and, of course, food remains. We refer to this condition as the *ecology inside a vial*. In recent years, we have been particularly interested in the analysis of the host trophic ecology, but the same samples are amenable to studies of different trophic levels (see Montagna et al. 2015, for a pioneering study on leaf beetle microbiomes, for instance).

PCR-based molecular characterisation of a predator’s food can be challenging, particularly in the case of carnivorous animals, when their food can belong to a closely related taxon, requiring a selective procedure to distinguish (and avoid) template DNA from the host. In a DNA metabarcoding framework, this can be achieved by using primers specifically designed to target a specific taxonomic group of potential diets (e.g., Riaz et al. 2011). One such example is the use of insect-specific mtDNA PCR primers to identify insects preyed by spiders, which takes advantage of the high mtDNA divergence between these two taxonomic Classes, allowing for selective PCR (e.g., Northam et al. 2012; Sint et al. 2015). Yet, even large taxonomic gaps could result in non-trivial technicalities hampering the design of suitable primers. However, the same type of analysis to investigate the diet of a herbivore is much simpler methodologically, since Nature provides already with the best possible tool: plastid DNA (cpDNA), exclusive of plants, and together with ITS sequences, the marker of choice for DNA-based plant identification, as well as for plant DNA-barcoding (Kress et al. 2005). Botanical molecular systematic research has provided through the years with robust universal primers targeting a variety of cpDNA loci to assist plant species diagnosis. Among these, two loci in particular have been selected as the standard for plant DNA-barcoding, the tandem *rbcL* and *matK* (CBOL Plant Working Group 2009). These and other loci are generally easy to amplify with specific and reliable universal primers which are not interfered, by definition, by animal DNA; they produce PCR fragments of suitable size for easy amplification and sequencing; and their continued use by botanists determines a high taxonomic representation in nucleotide sequence databases, which makes them suitable for identification purposes. While DNA-barcoding has favoured the use of length-invariant, protein coding loci, in our implementation for herbivore diet inferences, we have opted instead for length-variable cpDNA intergenic spacers, specifically the so-called *trnL* intron and most recently the *psbA-trnH* spacer (Jurado-Rivera et al. 2009; Gómez-Zurita and Cardoso 2014; Papadopoulou et al. 2015; De la Cadena et al., 2016). Sequence length differences are a nuisance for similarity assessment and genetic distance estimation and thus impair reliability of fast algorithms for taxonomic assignment. Yet, in our opinion, and specifically from a phylogenetic perspective, sequence length differences can be efficiently treated with current multiple sequence alignment algorithms, and provide with two main advantages: (1) they become an additional source of useful variation to increase the diagnostic value of these markers, and (2) size differences usually enable resolving homologous PCR products from different species by means of agarose gel electrophoresis, allowing to skip expensive and time-consuming cloning steps when studying the diet of leaf beetle individuals that fed upon two or more plant species.

We showed that this methodology is efficient and highly informative based on our extensive study of diets of Australian Chrysomelinae (Jurado-Rivera et al. 2009). In that study, we used *trnL* sequences obtained from whole specimen DNA extractions to infer the diet of 76 species in 24 genera of Chrysomelinae based on individual phylogenetic analyses carefully including all closely related homologous sequences available in GenBank at the time. In this proof-of-principle study, we were able to infer the correct host plant family in every case (for many species we had known host records), although resolution dropped at lower taxonomic levels (83% at tribal, and 51% at generic levels). Robust phylogenetic analyses provided a sound identification shortcut relying on information available in public sequence databases, and despite lower accuracy at infrafamilial taxonomic levels, we could refine our inferences, sometimes down to the species level, thanks to detailed floristic catalogues for the areas where the beetles had been collected. Yet public database incompleteness is a severe problem and inference power greatly benefits from availability of a local reference database for meaningful comparisons (e.g., García-Robledo et al. 2013). Indeed, since 2008 we have been working on setting a standard for this type of analyses whereby the analysis of the leaf beetle community goes hand-in-hand with a systematic compilation of angiosperm sequence data from the biome of interest to provide with a sound reference library for DNA-based inference of ecological associations. In the particular case of Nicaragua, we have sampled, vouchered, sequenced and made available to the scientific community *psbA-trnH* sequence data for some 450 plant species, nearly half of the plant diversity present in the Nicaraguan dry biomes, in an ongoing effort to enhance DNA-based species identification that we can use to characterise these valuable environments (Papadopoulou et al. 2015).

These approaches are becoming standard in many studies of tropical biodiversity, including studies on leaf beetles (Table 2) and other groups of phytophagous beetles, mainly weevils (Pinzón-Navarro et al. 2010; Kitson et al. 2013). But precisely in the context of large-scale and rapid biodiversity assessment, the generalisation of this type of studies is generating a new challenge. In our specific study of dry tropical forest structure and interactions in Nicaragua, we have analysed some 840 individual leaf beetle specimens, which yielded nearly 1100 sequences of putative diets. Such a large amount of data is not anymore amenable to individualised tree-based inferences, and two alternatives stand out to scale-up accelerated biodiversity assessment: either giving up trees and using fast BLAST-based approaches or, alternatively, automating the inference process. Given our concerns about the unavoidable problem of incomplete reference databases, especially when working at a regional scale or above, we have opted for the latter. Automated taxonomic identification from multiple sequences can be efficiently tackled by splitting the data into phylogenetically robust datasets together with taxonomically-tagged homologs from GenBank and/or a local reference database of known taxonomy. Making this procedure fully automated meets two main challenges: one is extracting this meaningful subset of homologs and their taxonomically relevant information, and the other is parsing phylogenetic trees for taxonomic information. We have developed a dynamic procedure that solves these problems in efficient ways to iteratively generate tree-based taxonomic identifications from large collections of unidentified DNA-barcoding data, which we called BAGpipe (‘Pipeline for Biodiversity Assessment using Genbank data’; Papadopoulou et al. 2015). Starting from a collection of sequence data of the selected genetic marker, the procedure uses a combination of local and global similarity searches to pick up all similar and putatively homologous sequences available in the latest Genbank release, recording their taxon ID and associated taxonomic hierarchy. At the same time, sequences are reoriented if needed, their ends trimmed to the length of the marker of choice, and redundant sequence data (i.e., population data) removed. These ensemble data constitutes the basis for subsequent phylogenetic matrix assemblage and phylogenetic inference, the so-called *reference database*. Robust phylogenetic inference is achieved for a certain level of sequence divergence where positional homology assessment is not compromised and homoplasy due to saturation is low (Goldman 1998; Yang 1998). Thus, we solved the problem of data partition for meaningful phylogenetic inferences by first splitting the unidentified *query sequences* in groups of similarity below a custom divergence threshold, each one used in turn to extract similar sequences from the reference database based on the same criterion. Query sequences and taxonomically identified reference database sequences within a predefined divergence threshold are submitted to multiple sequence alignment and maximum likelihood tree inference (and node support assessment). Automatically drawing taxonomic conclusions from trees was a challenge that we met exploiting the taxonomic hierarchy attached to Genbank data (inspired by Hunt and Vogler 2008; Chesters and Vogler 2013). The obtained unrooted trees are secondarily polarised and the most inclusive supported clades including unidentified query sequences are recognised, parsing the common taxonomy from reference sequences (i.e. Genbank taxon IDs and their hierarchy). This taxonomic inference, at the lowest taxonomic level allowed by the reference, is finally linked to the unidentified query sequence(s) using both strict and liberal criteria. In this context, it becomes obvious that coverage and reliability of available barcode reference libraries are critical for a meaningful use of this approach (Jinbo et al. 2011). A tool like BAGpipe (http://www.ibe.upf-csic.es/SOFT/Softwareanddata.html) makes it possible to boost large-scale biodiversity assessment both in its inventorying angle, but also in the study of interactions if applied to the identification of ecology-in-a-vial associations or metabarcoding studies.

**Table 2.** Molecular analyses of insect-plant associations for tropical Chrysomelidae.

Leaf beetle	Source	cpDNA marker	Host-plant	Reference
*Alagoasa decemguttata*	Nicaragua	*psbA-trnH*	Verbenaceae, Bignoniaceae	De la Cadena et al. (2016)
*Anadimonia* sp.	Borneo	*rbcL*	Lauraceae, Dipterocarpaceae	Kishimoto-Yamada et al. (2013)
*Arsipoda geographica*	New Caledonia	*trnL*	*Ardisia* (Myrsinaceae)	Gómez-Zurita et al. (2010)
*Arsipoda isola*	New Caledonia	*trnL*	Ericaceae	Gómez-Zurita et al. (2010)
*Blepharida suturalis*	Nicaragua	*psbA-trnH*	Burseraceae, Boraginaceae	De la Cadena et al. (2016)
*Brachycoryna pumila*	Nicaragua	*psbA-trnH*	*Sida* and *Triumfetta* (Malvaceae), *Chiococca* (Rubiaceae)	Papadopoulou et al. (2015)
*Calligrapha thermalis*	Mexico	*psbA-trnH*	*Perymenium* (Asteraceae)	Montelongo and Gómez-Zurita (2013)
*Cephaloleia* spp.	Costa Rica	ITS2, *rbcL*	Heliconiaceae, Zingiberaceae, Costaceae, Marantaceae, Cannaceae	García-Robledo et al. (2013)
*Chelobasis bicolor*	Costa Rica	*rbcL*	*Heliconia* (Heliconiaceae)	García-Robledo et al. (2013)
*Chelobasis perplexa*	Costa Rica	*rbcL*	*Heliconia* (Heliconiaceae)	García-Robledo et al. (2013)
*Dematochroma cancellata*	New Caledonia	*psbA-trnH*	Primulaceae, Lamiaceae, Millettieae (Fabaceae), Cunoniaceae, *Syzygium* (Myrtaceae), Sapindoideae (Sapindaceae)	Gómez-Zurita and Cardoso (2014)
*Glenidion* sp.	Nicaragua	*psbA-trnH*	Burseraceae, Fabaceae, Lantaneae (Verbenaceae)	De la Cadena et al. (2016)
*Heterispa vinula*	Nicaragua	*psbA-trnH*	Malvaceae, Cucurbitaceae, Annonaceae, Poaceae, Boraginaceae	Papadopoulou et al. (2015)
*Hyphaenia* sp.	Borneo	*rbcL*	polyphagous (7 plant families)	Kishimoto-Yamada et al. (2013)
*Liroetiella antennata*	Borneo	*rbcL*	Acanthaceae, Fabaceae, Fagaceae, Moraceae	Kishimoto-Yamada et al. (2013)
*Monolepta* spp.	Borneo	*rbcL*	polyphagous	Kishimoto-Yamada et al. (2013)
*Omophoita octomaculata*	Nicaragua	*psbA-trnH*	*Stachytarpheta* (Verbenaceae), Lamiaceae	De la Cadena et al. (2016)
*Parorectis rugosa*	Nicaragua	*psbA-trnH*	*Physalis* and *Solanum* (Solanaceae), Lamiaceae, Cucurbitaceae, Scrophulariaceae, Fabaceae	Papadopoulou et al. (2015)
*Physonota alutacea*	Nicaragua	*psbA-trnH*	*Cordia* (Boraginaceae), Fabaceae	Papadopoulou et al. (2015); De la Cadena et al. (2016)
*Platymela cephalotes*	Australia	*trnL*	*Acacia* (Fabaceae)	Jurado-Rivera et al. (2009)
*Syphrea* sp.	Nicaragua	*psbA-trnH*	*Acalypha* (Euphorbiaceae)	De la Cadena et al. (2016)
Taophila (Jolivetiana) mantillerii	New Caledonia	*psbA-trnH*	Polypodiopsida	Gómez-Zurita and Cardoso (2014)
Taophila (Lapita) spp.	New Caledonia	*psbA-trnH*	Cyatheales, Fabaceae, *Syzygium* (Myrtaceae), Rauvolfioideae (Apocynaceae), Oxalidales, Sterculioideae (Malvaceae)	Gómez-Zurita and Cardoso (2014)
*Taophila* s. str. spp.	New Caledonia	*psbA-trnH*	Polypodiopsida, Primulaceae, Millettieae (Fabaceae)	Gómez-Zurita and Cardoso (2014)
*Theopea* sp.	Borneo	*rbcL*	polyphagous (10 plant families)	Kishimoto-Yamada et al. (2013)
*Walterianella venustula*	Nicaragua	*psbA-trnH*	Lamiaceae, *Buddleja* (Scrophulariaceae)	De la Cadena et al. (2016)

### 2.4. Simultaneous progress in inventory and interactions

From our previous account, it should be clear already that the use of DNA has the potential to enhance simultaneously the study of both species inventories and species interactions, by using a limited number of standard laboratory and analytical techniques. In molecular systematics research, it is routine to use the PCR technique and suitable sets of primers to amplify more than one molecular marker from each sample. These data combined inform on the organisation of diversity and can potentially hint at specific evolutionary processes that shaped this diversity. Based on this common practice, we have easily incorporated to the lab routine the characterisation of a plant cpDNA marker from leaf beetle DNA extractions, in addition to our standard beetle markers. As a result, we systematically add a new ecological dimension to the description of diversity. We described several new tropical leaf beetle species interpreting DNA differences with other known beetle taxa, providing also with a DNA-based diagnosis of plant species for putative diet sequences. These include a southern Nearctic Chrysomelinae, the Mexican *Calligrapha thermalis* Gómez-Zurita associated to the composite *Perymenium mendezii* (Montelongo and Gómez-Zurita 2013), two species of New Caledonian Alticinae in the genus *Arsipoda* with one of them associated to Myrsinaceae (Gómez-Zurita et al. 2010), and two species of the New Caledonian endemic genus *Taophila* (Eumolpinae) together with an assessment of their dietary breadth (Gómez-Zurita and Cardoso 2014).

The above examples do not fall of course in the category of large-scale biodiversity assessment, although at least in the particular case of the study on the genus *Taophila*, it is a direct consequence, a refinement of findings derived from the wider biodiversity scope facilitated by this methodological approach (Papadopoulou et al. 2013). Nevertheless, each of these studies contributes individually to our understanding of tropical biodiversity and, if this strategy became the standard for systematic research in herbivore beetles, it would represent a fast progress in the complementary analysis of species and interactions. As seen, scaling-up this strategy for community analyses is feasible. Yet, we strongly believe that, even if some steps in species delimitation and identification are facilitated by the use of the described techniques, there will always be a dramatic need of taxonomic expertise to come full circle in any attempt for reliable biodiversity assessment.

## 3. Concluding remarks

As a short summary of our contribution, we can highlight that biodiversity is more than just species lists, and that biodiversity assessment should not neglect the way in which species are inter-connected in the ecosystems. Cataloguing biodiversity at large is certainly challenging, but it is also feasible, and DNA is possibly the key to fast and as comprehensive as possible inventorying of life forms, but also of their interactions. Phylogenies provide a robust approach to species delimitation and, in the absence of a comprehensive reference for comparison, the most robust approach to DNA-based species identification. Finally, the use of DNA as standard for species delimitation and identification makes these processes fully automatable, which is essential for high-throughput biodiversity assessment.

We tried to be constructive and discuss solutions to some of the current challenges in large-scale biodiversity assessment, however some fundamental problems remain and are not exclusively conditioned by technological or conceptual advancement. Rather, societal awareness (which is in great part our responsibility as professionals of biodiversity) and commitment of politicians and funding agencies alone can provide already a quantitative advantage for biodiversity research. As noted before, the emphasis for effective biodiversity research needs to be put again on funding expeditions and environmental sampling, pretty much with the same spirit as in the original voyages of discovery, but with the benefits of technology and trained specialists in different groups. Initiatives of this kind exist, most notably targeting insular systems, e.g. SANTO 2006, targeting the island of Espiritu Santo, the largest in the archipelago of Vanuatu (http://www.santo2006.org), or the Mo’orea Biocode Project, on the homonym island in the Tahiti archipelago (http://mooreabiocode.org). While these initiatives exceptionally mobilise millions of dollars and hundreds of scientists for comprehensive biological prospection, and are built with the right spirit, they typically yield a very modest global output. The reason is the currently existing bottleneck of available taxonomic expertise for extracting meaningful biodiversity information from these surveys, which remains the most serious challenge for large-scale biodiversity research (Kim and Byrne 2006). This towering limitation impairs not only the rigorous assessment of biodiversity in classical ways, but also our chances to count with a reliable taxonomy attached to public sequence databases, one of the most valuable resources for improving and speeding-up biodiversity assessment. Again, this challenge can be in part solved by restoring the importance and value of taxonomic research and allocating resources to taxonomic training, coupled with commitment and pedagogy for and from taxonomists to reinforce and expand available expertise.

Besides these fundamental limitations, there are still others of technical and conceptual nature which need to be dealt with, such as devising creative and efficient ways to incorporate new technologies for the improvement of large-scale biodiversity assessment. These should include for instance the use of next-generation sequencing technologies and environmental metagenomics, or more specifically in the case of insects the recently developed ‘metagenome skimming’ approach (Andújar et al. 2015; Linard et al. 2015), which promises to transform the standards of DNA-based biodiversity assessment by eliminating the PCR step and associated biases. Additionally, new automated procedures are required to democratise both species delimitation and identification (through reliable publicly available references). Of course, as conveyed in this assay from the start, there must be also a dedicated effort to routinely integrate ideas of inventory and interactions in biodiversity surveys, with an ever larger and more integrative scope. These and many more ideas are in the agenda of biodiversity researchers, as evidenced by many international Biodiversity Initiatives throughout the world and at different scales, of which these with global scope are the paradigm for large-scale biodiversity assessment, e.g. Global Ocean Biodiversity Initiative (http://www.gobi.org), Center for Tropical Forest Science (http://www.ctfs.edu), or the Global Genome Biodiversity Network (http://data.ggbn.org/index.php), among others. These initiatives address some of their objectives by bringing genomics, taxonomy and ecology together through the combination of strategic sampling and massive sequencing technologies, when possible.
